# KIMEHS—Proposal of an Index for Qualitative Evaluation of Children’s Menus—A Pilot Study

**DOI:** 10.3390/foods9111618

**Published:** 2020-11-06

**Authors:** Ada Rocha, Claudia Viegas

**Affiliations:** 1 Faculty of Food Science and Nutrition, University of Porto, 4150 180 Porto, Portugal; adarocha@fcna.up.pt; 2 Lisbon School of Health Technology, Polytechnic Institute of Lisbon, 1990-096 Lisboa, Portugal

**Keywords:** children’s meals, meals quality index, child obesity, children restaurant’s menu, food environment

## Abstract

Considering the importance of the food environments for health promotion, and the lack of simple, easy to use, low-cost measures of the quality of meals, the authors developed a qualitative menu index (KIMEHS—Kids’ Menu Healthy Score), tailored to children’s menu evaluation. Development of the tool was based on the Mediterranean food pattern. It includes 18 components, divided into seven main groups that reflect key aspects of menu quality, including protein source, side dishes, vegetables, dessert and beverages, and also allergens and nutritional information. The index was analysed for content and construct validity, as well as inter-rater reliability, and was applied to a sample of menus from restaurants in shopping centres in the Lisbon region. Possible index point ranges from −17 to 17, with a higher score indicating greater compliance with the recommendations. A value of 5.5 is obtained if all KIMEHS items are available, considering healthy and non-healthy options. The inter-rater reliability was assessed and values above 0.80 were obtained for Alpha Cronbach, as well as agreement % rate >75%. Agreement percentage is above 75% for all the components. Evaluated restaurants scored from −14 to 7, with an average KIMEHS of −6.15. Only four restaurants scored positive values, ranging from 0.25 to 7. KIMEHS was considered to be an adequate index to evaluate children’s menus, from the menu information displayed on restaurant websites and/or on restaurant displays or table menus. It is a simple, low-cost tool that may be used as a reference for health professionals as an objective measure to evaluate the food environment. Stakeholders could also be involved in their own assessment to help educate consumers about healthy food choices, strengthening the efforts to promote an adequate food pattern and health, contributing to the fight against obesity.

## 1. Introduction

Families eat frequently at restaurants [1]. The consumption of foods outside of home has been associated with unbalanced food patterns [2]. Evidence suggests that, when eating at restaurants, compared to when they eat at home, children consume more calories, total fat and saturated fat [3,4,5,6,7]. Moreover, they also consume sugar-sweetened beverages more frequently while eating at restaurants [2,3]. As families increase the consumption of food from restaurants, either table or fast-food meals, evidence suggests that the availability of healthy restaurant options specifically addressed to children influence children’s dietary intake and, consequently, their health. Restaurants have menus specifically tailored to children, but with limited availability of healthy options [3,8,9,10]. Obesity and poor diet quality are major public health concerns and the increase in food consumption outside the home is a major contributor [11].

However, we still do not have a clear picture of the food offer to children in restaurants, mainly due to the lack of comprehensive, standardised, and/or validated measures [3]. Some of the current tools include bromatological analysis to calculate meals’ nutritional content. Despite the fact that these are very objective methods, they reduce food to their nutrient composition, ignoring relevant aspects such as nutrient sources, and other qualitative characteristics. They are also time consuming and expensive.

Four qualitative approaches that aim to evaluate the quality of menus in food services were identified. The Menu Checklist was developed by Cassady et al. (2004) aiming to respond to the need for measures of influence of health promotion, including the restaurant environment. It was developed to be used either by consumers to help with health choices, or by technicians to collect information about the offer and consequently use results to increase awareness of restaurant owners. It could also be used by researchers to assess the healthfulness of restaurants and guide interventions for healthy eating availability and also to evaluate changes over time [12].

The Nutrition Environment Measures Study in Restaurants (NEMS-R) tool was developed to assess not only the food offered, but also the food environment that influences food choices. This tool includes facilitators of healthy eating (providing nutrition information), dishes labelled as healthier (e.g., low fat, low calories), offers of reduced-size portions, encouragement of menu changes (e.g., substitution of chips for vegetables), and availability of salad bar; in addition, barriers to healthy eating, such as encouragement for a larger portion, overeating or not allowing for special requests. The final calculation is performed including both quantitative (e.g., calories and saturated fat) and qualitative items (e.g., fruit without added sugar, whole grains) and also the price policy publicity techniques [13,14].

The third tool identified was the Children Menu Assessment (CMA), which is an expansion of the previous one, considering additional information addressed to children’s menus. It includes 29 items, 21 about the menu and eight related to the restaurant [3]. Both this tool and the NEMS-R use U.S. standards, namely, the 2010 Dietary Guidelines for Americans and the U.S. Department of Agriculture’s as reference [3,13].

Bernardo et al. (2013) proposed a healthy Dietary Diversity Index (DDI) to evaluate meals in self-service restaurants in Brazil. The DDI-M consists of four groups and 11 subgroups. The criteria for assessing the dietary diversity of a main meal were identified for each food group. The index ranges from −3 to +12, and includes cooking methods and nutrient density of foods, representing an observational, more qualitative approach. A higher dietary diversity score is associated with the presence of rice and beans, fruits, vegetables, lean meats and fish on the plate. It is used to define the food intake characteristics of diners who eat at self-service restaurants and may be used both to assess consumption and develop health promoting initiatives [15].

Researchers intended to evaluate the food offer addressed to children in restaurants, by reviewing menus available in the restaurants’ websites or in restaurants’ display and table menus. The previously described tools, although interesting and useful, were not applicable to the type of analyses required. Therefore, using the Mediterranean Food pattern, the authors decided to develop a qualitative menu index (KIMEHS—Kids’ Menu Healthy Score), tailored to children’s menu evaluation and apply it to a sample of Portuguese children’s menus.

## 2. Methods

### 2.1. KIMEHS Development

The development of this index was inspired by the need to evaluate menus from a qualitative perspective. Nutrition studies about food availability focus on macronutrient evaluation from bromatological analysis, which are time and cost consuming. Furthermore, this type of analysis focuses only on nutrient range compliance and do not consider the food that is being served, making it possible for a menu to fall within nutrient recommendations, but not within food portions guidelines [16].

Looking for a qualitative methodology to evaluate children’s menus, the authors identified other previously developed indexes, nevertheless none of them responded to the specific needs of this type of evaluation. Most indexes focus on restaurant evaluation rather than menu. Therefore, the proposed index was developed by nutritionists, aiming to be used by nutrition professionals and other health related technicians, to quickly evaluate the quality of a menu. Although it was developed to address children’s menus, it can be used with other types of menus.

#### 2.1.1. The Mediterranean Food Pattern and the Portuguese Food Guide

The Mediterranean Food pattern is widely recognised as healthy and sustainable [17,18,19,20,21,22]. Its principles are aligned with the Portuguese Food Guide recommendations [23], with a focus on a plant-based diet, with a higher intake of vegetables, cereals, pulses, moderate intake of fish and dairy products and low intake of meat, especially red and processed meat. This food pattern also highlights olive oil as the best fat source and the importance of moderate red wine intake [24,25]. These two items were not taken into consideration for the development of the KIMEHS due to the fact that it is not possible to evaluate the use of olive oil in the description of the menu and alcoholic beverages are not recommended for children’s consumption.

Since the recommendations from the Portuguese Food Guide are set to ensure adequate nutrient intake, the components of the KIMEHS include all of the major food groups, namely, non-starchy vegetables, cereals and starchy vegetables, pulses, meat, fish and eggs, fruit and water (Table 1). Exceptions were made for dairy products, which are not usually a part of main meals in Portugal, and also fats, due to the fact that it is not possible to retrieve this information through the description of the menu.

#### 2.1.2. KIMEHS Evaluation Scores

The KIMEHS is a tool developed by the researchers to evaluate the extent to which a kids’ menu is consistent with recommendations for children, considering the Mediterranean food pattern (consumption of vegetables, cereals and starchy vegetables, pulses, fruit, fish and water; limiting the consumption of processed and red meat as well as sugar sources).

This index includes 18 components, divided by 7 main groups, that reflect key aspects of menu quality, including protein source, side dishes, vegetables, dessert and beverages, and also allergens and nutritional information.

Each menu component is evaluated individually, and the score is attributed according to the degree of compliance. The main groups of the KIMEHS are represented in Table 1. Positive points are attributed to healthy options on the menu, while non-healthy choices are negatively scored. The magnitude of the attributed points is proportional to the impact that the food option has on menu quality and health. From the KIMEHS components, protein sources are divided in 8 sub-items that evaluate the type of protein source (animal vs. vegetable, and among animal proteins, the cooking method, also including an item for processed meat). The attributed score for each item is set according to the hierarchy of the recommendation and nutrient composition. Fish, being of a high biological value and a sustainable source of protein, is scored with 2 points; pulses are scored 1.5, due to their high sustainability, although they are low biological value protein; lean meat, being less sustainable than the previous two, is scored 1 point, and red meat, due to its higher content of saturated fat is scored 0.5 points [26,27]. Negative points are attributed to the presence of fried items or processed meat such as sausages, nuggets, bacon, fish fingers, etc. Taking the example of fried potatoes, its presence in the menu is scored negatively with −2 points while its absence is positively scored with 1 point. After evaluation of all the components, a sum of each score is calculated, giving the final KIMEHS. Additional components were created to include nutritional and allergen information.

Side dishes usually include potatoes and cereals. One point is attributed to rice, pasta, bread, corn and potatoes. Two negative points were attributed to the presence of fried potatoes.

No criteria were defined for whole grains due to the fact that it is not possible to retrieve this information through menu description.

Vegetable consumption is recognised essential for a sustainable and healthy lifestyle, to ensure fibre, vitamins and minerals intake. They are also important for the prevention of several chronic diseases, such as cardiovascular diseases, certain types of cancer, diabetes and obesity. Their high nutrient density is associated with a low energy intake, contributing to satiety and weight management [28,29]. Vegetables represent an important part of the Mediterranean Food pattern and a significant group in the Portuguese Food Guide [22]. Therefore, a score of 2 was attributed to the presence of vegetables either in soup or as an individual component. In order to enhance their importance, a score of −2 is attributed to their absence.

Desserts may include fruit and/or sweet desserts. Following the recommendations, in order to promote fruit instead of desserts with added sugars and fat, 2 points are attributed to the presence of fruits, while 2 negative points are attributed to their absence and the opposite is valid for sweet desserts, with a negative point attributed to their presence and a positive point attributed to their absence.

The same assumption was used for beverages, for which water was considered to be the recommended drink. Two positive points are attributed to its presence and two negative points attributed to its absence. Sugary drinks, such as fruit juices and soft drinks get a negative point, while their absence gets a positive point [30].

The availability of nutritional and allergen information was positively scored with 0.25 points each. However, no penalty was applied if this information is not available.

Possible index scores range from −17 to 17; negative scores are related to poor food options on the menus and higher scores indicate greater compliance with food recommendations. A value of 5.5 is obtained if all score items are available, including both healthy and unhealthy options.

#### 2.1.3. Content Validity and Reliability

Content validity of the KIMEHS was supported by an extensive review, including the Portuguese Food Guide, the Mediterranean Food pattern [23], and also the Portuguese Guidelines for School Meals [31], to confirm that KIMEHS components reflect all the main recommendations for children’s meals. The index includes all the major food groups, as described in the previous paragraph.

Considering previous research from other developed indexes [12,13], two types of analysis were made to validate the index. As found in similar research, first, model children’s menus, developed by nutritionists were analysed for construct validity, to prove that the KIMEHS reflects the theoretical concept of a high-quality menu [32]. These menus were retrieved from the Portuguese General Directorate of Education [31].

Secondly, assessment of inter-rater reliability was achieved by conducting a total of three complete assessments of each menu, as reported in a previous research [13]. For this purpose, three researchers were given the KIMEHS (Table 1) and an Excel spreadsheet previously prepared with the index scores and calculations. The index was briefly explained using two menu examples, using an Excel spreadsheet. The researchers then received 44 different children’s menus that were independently and completely analysed. Inter-rater reliability was assessed by percent agreement and alpha-Cronbach coefficient. Alpha Cronbach values above 0.80 were considered high [33], as well as agreement % rate >75%.

### 2.2. KIMEHS Applicability

After the development of the index, authors applied it to the identified menus, in order to comprehend whether the index was useful and returned values that would reflect the quality of the menus.

#### Menu Selection and Information Collection

All restaurants from 16 shopping centres (all shopping centres from the Lisbon region) were selected in order to apply and test the developed index. A total of 884 were available, including repeated restaurants, from these 184 represented different sit-down and fast-food restaurants, from which 44 supplied children’s menus that were evaluated. These menus were collected from websites and physical locations and evaluated using KIMEHS.

### 2.3. Data Analysis

KIMEHS was computed using Microsoft Excel version 16. Data analysis was performed using R version 4.0 to calculate Cronbach alpha and Pearson correlation. Statistical significance was considered at *p* < 0.05.

## 3. Results and Discussion

### 3.1. Content Validity and Reliability

Analysis of children’s menus developed by nutritionists resulted in all menus receiving high scores (over 15 points). Menus from the Portuguese General Directorate of Education comply to strict guidelines about which foods should be supplied, frequency of certain foods and cooking methods, as well as restricting the offer of desserts and sweet beverages [31]. The high score results obtained from these menus demonstrate that the KIMEHS is able to reflect the high-quality nutritional concept of the menu.

Results from the independent assessment of three researchers that completely analysed 44 different children’s menus revealed a high inter-rater reliability, as well as a high agreement percentage among researchers. The KIMEHS total score has a high alpha (0.86) and a high agreement percentage (82%). For some of its components (availability of fried options and processed food, sugary drinks and desserts and nutritional information), the alpha is below 0.8, but still presents a good value (>0.75). The component that has a lower alpha is availability of water (0.6). Agreement percentage is above 75% for all the components—Table 2.

Both types of analyses demonstrate that the developed score—KIMEHS—can be considered to be an adequate index, to be used by nutritionists and health professionals, to evaluate children’s menus, from the menu information displayed on restaurant websites and/or available in restaurant displays or table menus, because its components were considered to have acceptable and very good inter-rater reliability.

### 3.2. KIMEHS Values for Evaluated Menus

**Figure 1:**
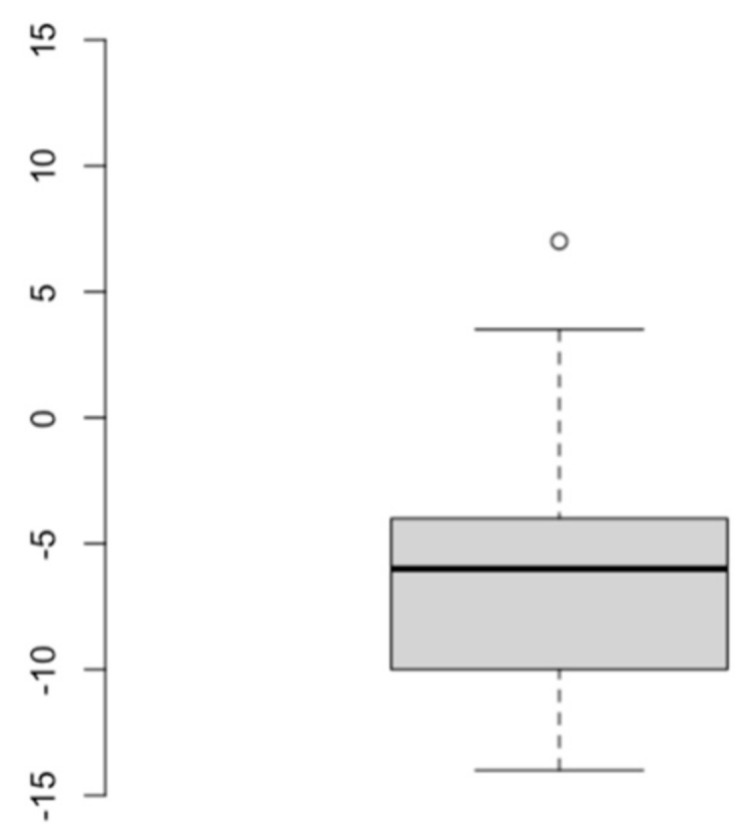
Score distribution of analysed children menus.

Evaluated restaurants scored from −14 to 7, with an average KIMEHS of −6.15. Only four restaurants scored positive values, ranging from 0.25 to 7. Figure 1 represents the boxplot illustrating the score distribution for the 44 analysed menus.

The scoring system is similar to existing tools, such as the NEMS-R that also attributes positive points to restaurants that provide menus based on the number of healthy entrees, salads, whole grains, healthy beverages, side dishes and desserts, while deducting points for the presence of soda, free refills on sugary beverages, unhealthy desserts, and the use of toys or other child-directed marketing [12]. However, while the NEMS-R is intended to evaluate restaurants, KIMEHS is directed at evaluating the menus.

The majority of evaluated menus score negatively on KIMEHS, implying that it is not possible to choose a healthy meal, mostly due to the absence of soup, vegetables, fruit and water. Fish is somewhat available, but most frequently fried. From menu evaluation, availability of bread was mostly associated with a hamburger offering in which fried potatoes are also part of the menu. These results are consistent with findings from other studies that also reported the lack of healthy menu options in children’s menus [34,35,36], frequently offering sugary drinks, food rich in saturated fat [35], sodium and calories [36,37,38].

Table 3 presents the percentage of availability of the previously presented KIMEHS items. The typical offer is characterised by both red and lean meat options, often fried, accompanied by fried potatoes, sugary drinks and desserts. Fish availability is usually processed and fried. Vegetables and pulses are absent from most menus and water, when available, is an option among sugary drinks. These results pair with the previously presented KIMEHS results that evidence the poor menu offering from all the evaluated restaurants.

Pearson correlations that were calculated between KIMEHS components demonstrate that negative scores are associated with the offer of fried options, lack of vegetables, fruit and pulses, while positive scores are associated with the opposite. Significant moderate to strong correlations were found between the KIMEHS and fish, vegetables and fruit (Table 4).

KIMEHS was designed to evaluate the options available in children’s menus, penalizing availability of less healthy options and benefiting the healthier ones. If all unhealthy items are available, positive KIMEHS (0.5) is attainable if at least two healthy items are also available. A KIMEHS of 5.5 is attainable, if all healthy and unhealthy items are available. Removing fried options from the menu will result in a KIMEHS of 11.5, and 13.5 if processed meat is also removed, both of which would be considered moderately healthy. High KIMEHS values would be above 13.5, which would require additional removal of sugary drinks and desserts.

When comparing this KIMEHS with previously designed indexes, the authors found this one to be easier and faster to use, returning a feasible quantitative approach of menu quality. NEMS-R was designed to evaluate the restaurant environment in general, while KIMEHS concentrates on the menu. The NEMS-R, within its evaluation, considers the availability of kids’ menus in restaurants and whether or not it provides a healthy option, based on a “yes” or “no” answer [13]. It does not return a score for this menu, while KIMEHS clearly intends to quantify the quality of the menu, allowing comparison between restaurants and menus. The Menu Checklist [12] intends to evaluate menus, but again in a qualitative perspective analysing whether healthy items are available or not, failing to return a quantitative measure of the quality of the menu. The approach that was found to be more intuitive and similar to KIMEHS was the DDI, designed to evaluate consumption attributing a score, according to the type of food, its energy density and preparation/cooking method [15]. Although this index was developed to evaluate food intake, it could be applied to menu evaluation. Nevertheless, the scoring system considers a different rationale, only attributing negative points to high energy density grains and derivatives, not including beverages, sweet desserts or availability of allergens and nutrition information, which makes KIMEHS a more complete comprehensive tool.

**Figure 2:**
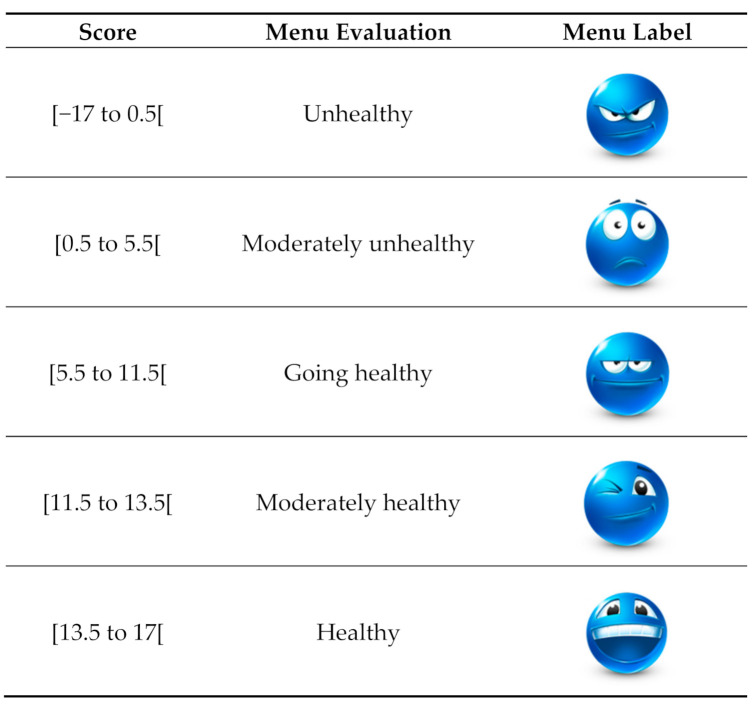
Proposal of simplified menu label based on the obtained KIMEHS. Icons credited to Emoticons Icons by ArtDesigner.lv, free for non-commercial use; commercial usage: allowed (backlink to http://artdesigner.lv required).

Although the index was developed to be used by nutritionists and health professionals as a tool for menu quality evaluation, the final score could be used as an indicator for consumers and restaurants of the healthiness of the menu, hence, act as a driver for change. There is a growing concern from individuals about food and health and food options at restaurants [39,40] and specifically focusing in children’s meals [4,5]. At the same time, some food chains have already tried to comply with nutrient recommendations aiming to respond to customers’ expectations [5,6,7,11]. From the public health perspective, acting on food environments is considered a fundamental strategy [41]. The use of this index by restaurants would need an adaptation, transforming the score into a more intuitive simplified visual scale to be read and understood by consumers. Therefore, the authors propose a menu evaluation based on the obtained score and a simplified menu label—Figure 2.

This index has some limitations. It does not distinguish the type of cereal (refined or non-refined cereals) used for bread, pasta or rice and, in spite of considering some items that contribute to increase energetic value, it does not account for the energetic value of the meals. Although it accounts for the presence of pulses, vegetables and fruit served separately (e.g., vegetables inside hamburgers are not taken into consideration), it does not consider the size of the portion served. It also does not consider the price of healthy options or other marketing techniques that may facilitate or impair the choice of a healthy option. KIMEHS is not yet suitable for vegetarian menus evaluation, mainly because it was developed considering the current reality of children’s menus. This adaptation can be done in the future. Another limitation of the observation method is that more healthful food offers, especially for buffet and sit-down restaurants, may be available than apparent on the menu or websites. Nevertheless, this tool intended to examine the menus designed specifically for children, usually referred to as “kids’ menu”, “for kids”, “bambino”, etc.

The use of the developed index for the previously described restaurant sample was a pilot study to validate KIMEHS, which will be used with a larger sample in the future.

## 4. Conclusions

The authors conclude that this index is easily applied, cost-effective, and does not imply a close interaction with restaurant owners to assess menu evaluation, as it uses only the information available on the displayed or offered table menus.

Considering the current scenario of children’s menu offerings, this index may be used as a reference for health professionals as an objective measure to influence health promotion, such as restaurant environment, but also involving the stakeholders in their own assessment. This could promote investments from restaurants in their menu planning, aiming for higher scores that can be used as a marketing tool to attract more clients. Moreover, it could help educate consumers about healthy food choices, while changing the food environment, strengthening the efforts to promote an adequate food pattern and health. The wide application of this simple, low-cost index by restaurants during menu planning, and by technicians when auditing and monitoring children menus, may be a major contribution to the fight against obesity.

## Figures and Tables

**Table 1:** Description of Kids’ Menu Healthy Score (KIMEHS).

Item	Standard for Positive Score	Score	Standard for Negative Score	Score
Protein source	Red meat	0.5	No red meat	0
	Lean meat	1	No lean meat	0
	Fried red meat	−1	No fried red meat	0
	Processed red meat	−2	No processed red meat	0
	Fried and/or processed lean meat	−1	No fried and/or processed lean meat	0
	Fish	2	No fish	−1
	Fried fish	−1	No fried fish	0
	Pulses	1.5	No pulses	0
Side dishes	Fried potatoes	−2	No fried potatoes	1
	Other (rice, pasta, bread…)	1	No other side dishes	0
Vegetables	Vegetables	2	No vegetables	−2
	Soup	2	No soup	−2
Dessert	Fruit	2	No fruit	−2
	Sweet dessert	−1	No sweet dessert	1
Beverages	Water	2	No water	−2
	Sugary drinks	−1	No sugary drinks	1
Allergens information	Provides	0.25	Does not provide	0
Nutritional information	Provides	0.25	Does not provide	0

**Table 2:** Menu components content and reliability.

KIMEHS Group Items	% Agree	Alpha (Cronbach)
KIMEHS	82	0.86
Availability of fried options and processed food	93	0.77
Availability of vegetables, fruits, pulses, cereals and starchy vegetables	85	0.85
Availability of water	84	0.60
Availability of sugary drinks and desserts	95	0.75
Availability of healthy meat and fish options	93	0.89
Allergen and nutrition information	77	0.76

**Table 3:** Availability (%) of KIMEHS components in evaluated menus.

KIMEHS Items	%
Protein source	
Red meat	43
Lean meat	39
Fried red meat	5
Fried and/or processed meat	39
Fried lean meat	30
Fish	27
Fried fish	20
Pulses	0
Side dishes	
Fried potatoes	50
Other (rice, pasta, bread…)	59
Vegetables	
Vegetables	11
Soup	11
Dessert	
Fruit	16
Sweet dessert	32
Beverages	
Water	32
Sugary drinks	50
Allergens information	18
Nutritional information	7

**Table 4:** Correlations of the KIMEHS components.

Component	KIMEHS	Red Meat	Fried Red Meat	Processed Meat	Lean Meat	Fried Lean Meat	Fish	Fried Fish	Fried Potatoes	Cerealsand NSV ^1^	Pulses	Vegetables	Soup	Water	Fruit	Juices	Sweet Desserts	Aller-gens	Nutritional Information
KIMEHS	1																		
Red meat	0.16	1																	
Fried red meat	0.07	−0.09	1																
Processed meat	0.30	0.10	0.00	1															
Lean meat	0.24	−0.02	−0.06	0.02	1														
Fried lean meat	−0.10	0.09	0.01	−0.01	−0.58	1													
Fish	0.47 *	0.01	0.04	0.03	−0.07	0.04	1												
Fried fish	−0.15	0.02	0.01	−0.06	0.04	−0.04	−0.61	1											
Fried potatoes	0.24	−0.17	−0.03	−0.06	−0.21	0.18	0.05	−0.08	1										
Cereals and NSV	0.34	0.06	0.08	0.14	0.10	0.08	0.05	−0.02	0.19	1									
Pulses	0.39	0.11	0.05	0.14	0.32	0.03	0.03	0.13	−0.14	0.22	1								
Vegetables	0.62 *	0.26	0.05	0.21	0.14	−0.12	0.18	0.00	−0.08	0.12	0.26	1							
Soup	0.19	−0.12	0.03	−0.02	0.08	−0.11	0.03	−0.11	0.09	0.00	−0.06	−0.04	1						
Water	0.20	−0.08	−0.03	−0.09	0.11	−0.06	0.02	−0.05	0.05	0.00	−0.06	0.02	0.14	1					
Fruit	0.53 *	0.18	0.08	0.14	0.31	−0.35	0.00	0.05	−0.09	0.15	0.28	0.24	0.10	0.00	1				
Juices	0.27	0.07	−0.06	−0.03	0.13	−0.02	0.04	0.13	0.01	0.08	0.21	0.06	−0.11	−0.44	0.17	1			
Sweet dessert	0.09	−0.04	−0.03	0.05	0.01	0.04	0.05	0.09	−0.13	−0.13	0.18	−0.07	−0.16	−0.24	−0.11	0.36	1		
Allergens	0.15	−0.04	0.09	−0.09	0.11	−0.09	−0.12	0.15	0.01	0.01	0.00	0.09	0.07	0.17	0.31	−0.05	−0.20	1	
Nutritional information	0.21	−0.12	0.08	−0.04	0.23	−0.21	−0.16	0.11	0.05	0.08	−0.03	0.09	0.11	0.24	0.41	−0.05	−0.19	0.72	1

* significant Pearson correlation (*p* < 0.01); ^1^ NSV—Non starchy vegetables.
